# Seasonal changes in morphology govern wettability of Katsura leaves

**DOI:** 10.1371/journal.pone.0202900

**Published:** 2018-09-27

**Authors:** Hosung Kang, Philip M. Graybill, Sara Fleetwood, Jonathan B. Boreyko, Sunghwan Jung

**Affiliations:** 1 Department of Biomedical Engineering and Mechanics, Virginia Tech, Blacksburg, VA 24061, United States of America; 2 Department of Mechanical Engineering, Virginia Tech, Blacksburg, VA 24061, United States of America; 3 Department of Material Science and Engineering, Virginia Tech, Blacksburg, VA 24061, United States of America; 4 Department of Biological and Environmental Engineering, Cornell University, Ithaca, NY 14853, United States of America; University of Vigo, SPAIN

## Abstract

Deciduous broad-leaf trees survive and prepare for winter by shedding their leaves in fall. During the fall season, a change in a leaf’s wettability and its impact on the leaf-fall are not well understood. In this study, we measure the surface morphology and wettability of Katsura leaves from the summer to winter, and reveal how leaf structural changes lead to wettability changes. The averaged contact angle of leaves decreases from 147° to 124° while the contact-angle hysteresis significantly increases by about 35°, which are attributed to dehydration and erosion of nano-wax. Due to such wettability changes, fall brown leaves support approximately 17 times greater water volume than summer leaves.

## Introduction

Leaves are an important part of a tree for photosynthesis, respiration, transpiration, and storage of chemical energy and water [1–3]. However, deciduous trees survive dry and cold winters most notably by shedding their leaves in fall [4]. To lose their leaves, trees undergo a number of physiological and morphological changes in the leaves, stems, and roots [5, 6]. Among these changes, an abscission layer at the base of a leaf’s petiole plays a critical role in facilitating the leaf fall process. The formation of the abscission layer during the fall cuts off water and nutrient transport through the xylem and phloem [5–8], which expedites the dehydration in the leaf [6]. This dehydrated abscission layer weakens cell adhesion between the leaf and stem, causing the leaf to fall from the tree more easily by wind or rain [6]. Additionally, leaves are found to be less hydrophobic in the fall [9].

The hydrophobic property of tree leaves has been studied extensively [10–13]. Hierarchical, two-tier roughness is a typical structure of hydrophobic leaf surfaces (e.g. Lotus leaf). The first tier is composed of epidermal cells that form convex bumps at the microscale and the second tier is tubular wax-nanocrystals. As a consequence of the surface structure, when a rain or dew droplet rolls on a two-tier hydrophobic leaf, particulate dirt is collected by the droplet and removed from the surface, so-called the “self-cleaning lotus effect” [10–12]. Previous studies have focused on the surface structure and wettability of nourished leaves, however it is yet to be understood how seasonal changes in a leaf’s structure affect its wettability.

Leaf wettability could affect the dynamics of an elastic leaf upon rain droplet impact. Droplet-impact experiments with an elastic structure similar to a leaf (cantilever beam beam [14, 15], fiber [16] and butterfly wing [17]) showed that the elastic structure vibrates and its initial displacement is roughly the same regardless of wettability. However, a higher torque is triggered at the base of a hydrophilic beam than that of a hydrophobic beam due to the adhered liquid [14].

In this study, we investigate how structural changes on a leaf surface modify the leaf’s wettability during seasonal change. Specifically, a functional relationship between the two-tier structures and the observed contact angles of the leaf’s surface will be found. First, micro- and nano-structures of *Cercidiphyllum japonicum* (Katsura) leaves were characterized using scanning electron microscopy (SEM), thereby characterizing morphological features such as two-tier roughness, areal fraction of the air/wax, and liquid/wax interfaces. Then, a combined Wenzel and Cassie-Baxter contact-angle model is proposed to take into account an inhomogeneous eroded area on a leaf surface, which is only observed in fall leaves. To further correlate different configurations of micro- and nano- structures to the surface wettability, Katsura leaves are treated in various conditions (vacuum, heat, and chloroform). We discuss the effect of low contact angle and high contact-angle hysteresis on a leaf, and a potential application to use the nano-wax tubules from leaves as a coating material.

## Materials and methods

### Study location and leaf samples

We selected the Katsura tree, *Cercidiphyllum japonicum*, for this study. Katsura trees are deciduous, which are native in China and Japan [18]. The leaves are heart-shaped and approximately 8 cm long and 6 cm broad when mature. The leaf petioles are short, only about 3 cm long. Generally, the leaves grow closely packed together. The mass and area of Katsura leaves (N = 9) are 430 ± 50 mg and 22 ± 5 cm^2^, respectively. SEM observations confirm that the tree is hypostomatous (i.e., stomata only appear on the abaxial leaf surface).

Katsura trees near Gilbert Street and at Virginia Tech’s Hahn Horticultural Garden in Blacksburg, VA (37°14’00.3”N, 80°25’17.1”W and 37°13’11.0”N, 80°25’26.9”W) were selected for study samples (N > 100). Blacksburg is in a humid continental climate zone with an average temperature range from 4.5 to 17.3°C annually. Annual precipitation, wind speed, and relative air humidity averages are approximately 1000 mm, 2 m/sec, and 76%, respectively [19]. For the purposes of this study, we classified the morphological phases of the leaves based on the season. Here, the spring leaves are the ones taken from April to May, spanning the time from when the leaves first begin to appear on the tree until the leaves lose their reddish-green color. The summer leaves are taken approximately from June to September. During the summer, the leaves are fully grown and dark green. The fall leaves are samples taken from October to November, and have either a yellow or brown color.

### Contact angle measurement

Contact angle measurements were performed using a tilting goniometer (Model 590; ramé-hart Instrument Co.) and DROPimage Advanced software. A 10 *μ*L water droplet at room temperature (≈25°C) was gently released on a leaf sample which is fixed to a petri dish with a double-sided adhesive tape [20]. Then, the sample with a water droplet was tilted by the goniometer to determine the advancing ($\theta_{\text{Adv}}^{*}$) and receding ($\theta_{\text{Rec}}^{*}$) contact angles as well as the critical tilting angle. The advancing and receding contact angles were measured just prior to the movement of the droplet.

### FE- or E-SEM images

FE-SEM (LEO 1550, Zeiss) was used to visualize and analyze topological changes on a leaf surface. First, a leaf was cut to exclude regions containing the midrib and veins. Then, the leaf sample was mounted to a plate using a double-sided carbon tape. Top-view images were taken at various magnifications. To measure the cross-sectional view, the leaves were immersed in liquid nitrogen for 10 sec and broken between two grips. Then, the sample plate was loaded into the SEM chamber.

We used ESEM (Quanta 600 FEG, FEI) to visualize the solid/liquid interface on papillose epidermal cells by detaching a frozen droplet water droplet from the leaf surface. The leaf was immersed into liquid nitrogen for 10 sec, and upon removal, a 10 *μ*L droplet was pipetted onto the leaf surface. Immediately afterward the leaf and droplet were placed in a Peltier-cooled specimen holder at -15 °C. The samples were mounted using a double-sided copper tape for high heat conduction. Once the ESEM pressure was increased to 666 Pa, the water droplet partially detached from the leaf surface as shown in S1 Fig. The chamber pressure condition for sublimation was over 133 Pa.

### Image analysis

We used image analysis methods to analyze SEM images of various leaf conditions. Customized Matlab codes were used to determine the surface roughness (*r*_micro_), the areal fraction of liquid/solid interface (*φ*_nano_), and the areal fraction of intact wax regions (*α*). First, to evaluate *r*_micro_, we need to track the edges of the epidermal cells from FESEM images. The images were converted into black and white images for the Matlab edge-detection algorithm. To determine *φ*_nano_, the image histogram was used to choose a reliable and systematic parameter in grayscale (details shown in S4 Fig). The areal fraction of intact wax regions (*α*) was approximated using different contrasts. We ignored wax regions less than 5 *μ*m^2^ in area and eroded wax regions less than 22.5 *μ*m^2^ in area. The image analysis results show the tracked boundary of the eroded regions as blue lines in S2(c) and S2(d) Fig.

### Lab condition treatment

Leaf samples were attached onto petri dishes with a double-sided adhesive tape prior to vacuum, heat, or chemical treatments. For the heat treatment, the leaf samples were placed in an oven (Thermo Scientific, HERATHERM oven) at 55, 65, and 75°C and we monitored the contact-angle variation (see S7 Fig). For the vacuum treatment, we placed leaf samples in a vacuum desiccator at 20 kPa for 24 hours. To remove wax, we tilted a leaf sample at an angle around 30 degrees and dripped 10 mL of a chloroform solution (Cambridge Isotope Laboratories Inc., Chloroform-D 99.8%). Then, the collected solution of chloroform and leaf wax is gently dried with nitrogen gas as shown in S5(a) Fig.

### Wetting state in nanoscale waxes on leaves

To prove the Cassie-Baxter state at the nano scale, the critical height at a given spacing of nano-wax tubules to transition from the Cassie-Baxter to Wenzel states will be estimated for green and brown leaves. We assumed the nano-wax tubules are vertically aligned and water contact only the tip of the wax and the contact angle on the tubules is the same as the advancing contact angle on a flat wax surface (*θ*_Adv_ = 117°) (See the schematics of S4 Fig) The spacings (2*d*_*wax*_) of green and brown leaves are measured to be approximately 75 ± 20 nm and 815 ± 350 nm, respectively. Then, the radius of curvature of the meniscus becomes *d*_*wax*_/sin(*θ*_Adv_−*π*/2). Using a simple geometric relation, we can estimate the penetration depth of the air/water meniscus, *h*, as

$$\frac{h}{d_{wax}}=\left[\frac{1}{\text{sin}\left(\theta_{\text{Adv}}-\pi /2\right)}-\sqrt{{\left(\frac{1}{\text{sin}\left(\theta_{\text{Adv}}-\pi /2\right)}\right)}^{2}-1}\right].$$ (1)

The calculated depth of green leaves is 25 nm, which is significantly shorter than the height (1.0 ± 0.2*μ*m) of nano-wax tubules in the summer. It means that a droplet on green leaves stays in the Cassie-Baxter state at the nanoscale. For brown leaves, the calculated depth is 277 nm longer than the height (134 ± 40 nm) of nano-wax tubules in the fall. As illustrated in the schematics of S4(b) Fig, the air/water meniscus will touch the bottom of the leaf surface, and a small volume of air pockets will remain only around sparsely distributed nano-wax tubules. Therefore, we can assume the Cassie-Baxter state for green leaves and the Wenzel state for brown leaves at the nanoscale.

## Results

### Changes in leaf structure and wettability over season

**Fig 1:**
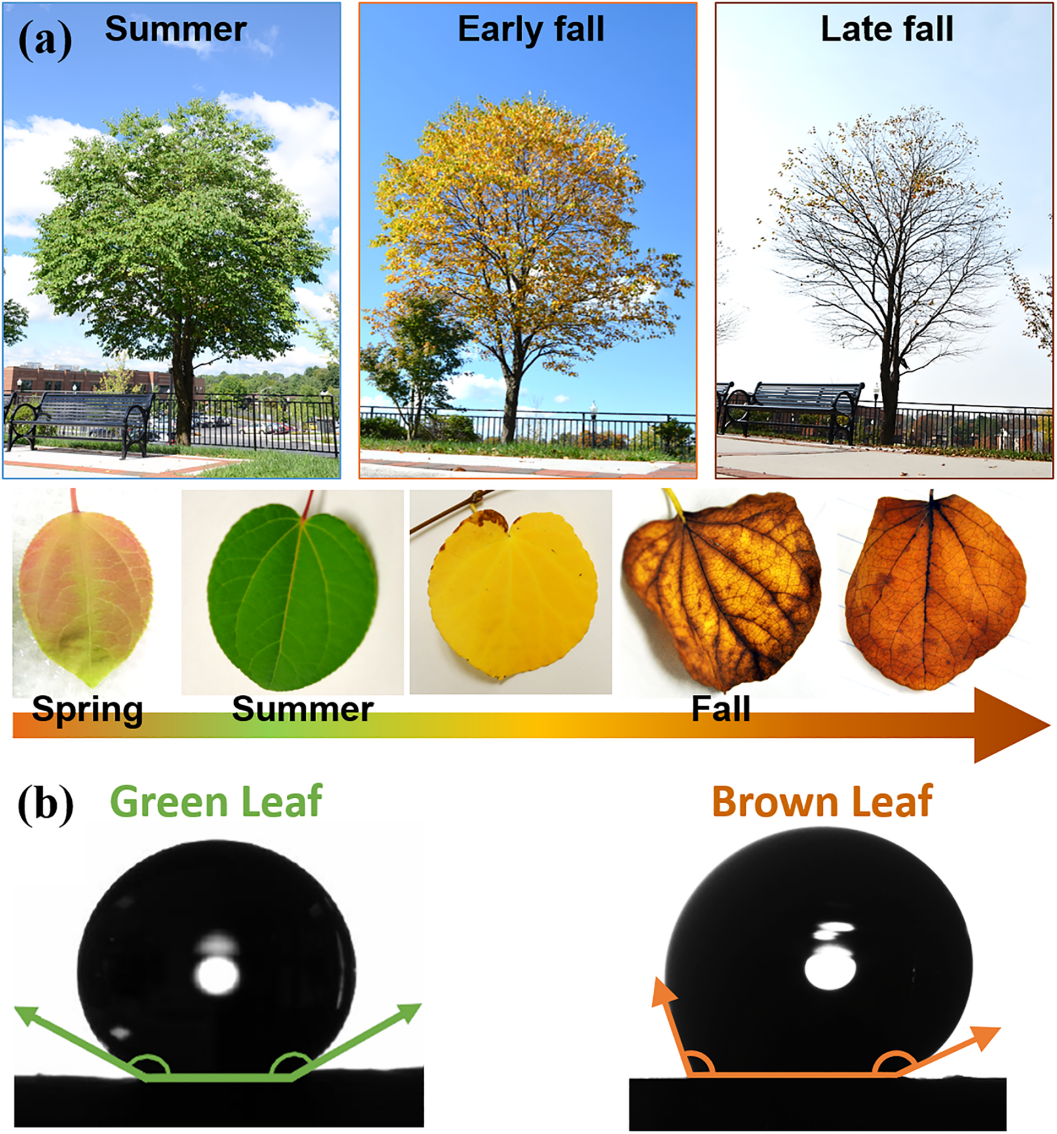
(a) Images showing the seasonal changes of a Katsura tree from spring to fall. The leaf color changes to reddish green—green—yellow—yellowish brown—brown. (b) Images of a sliding droplet on green and brown leaves to show the contact angles. The green leaf exhibits a superhydrophobic surface ($147{}^{\circ},\theta_{\text{Adv}}^{*}/\theta_{\text{Rec}}^{*}=157{}^{\circ}/137{}^{\circ}$) whereas the brown leaf becomes less hydrophobic ($123{}^{\circ},\theta_{\text{Adv}}^{*}/\theta_{\text{Rec}}^{*}=151{}^{\circ}/96{}^{\circ}$).

Fig 1(a) shows seasonal variations of a Katsura tree. In the spring, heart-shaped leaves emerge with a reddish-green color, later changing to green as they mature in the summer. In the fall, the leaves turn yellow and then brown. Not only does the leaf color change, the wettability also changes over the season. We measured both advancing and receding contact angles by tilting a sample substrate until a droplet is about to move (an inclined plate method; see details in “Materials and Methods”). The maximum and minimum values of contact angles are defined as the advancing contact angle ($\theta_{\text{Adv}}^{*}$) and receding contact angle ($\theta_{\text{Rec}}^{*}$), respectively. Our contact-angle measurements show that Katsura leaves change from superhydrophobic in the summer ($147{}^{\circ},\theta_{\text{Adv}}^{*}/\theta_{\text{Rec}}^{*}=157{}^{\circ}/137{}^{\circ}$) to sticky and hydrophobic in the fall ($123{}^{\circ},\theta_{\text{Adv}}^{*}/\theta_{\text{Rec}}^{*}=151{}^{\circ}/96{}^{\circ}$) as shown in Fig 1(b). The averaged contact angle has decreased by 24° while the contact-angle hysteresis has increased by 34°. The critical angles for a 10 *μ*L droplet to roll off for green and brown leaves are 12 ± 6°, and 70 ± 20°, respectively, which indicate that water wets a late-fall leaf better than a summer leaf.

**Fig 2:**
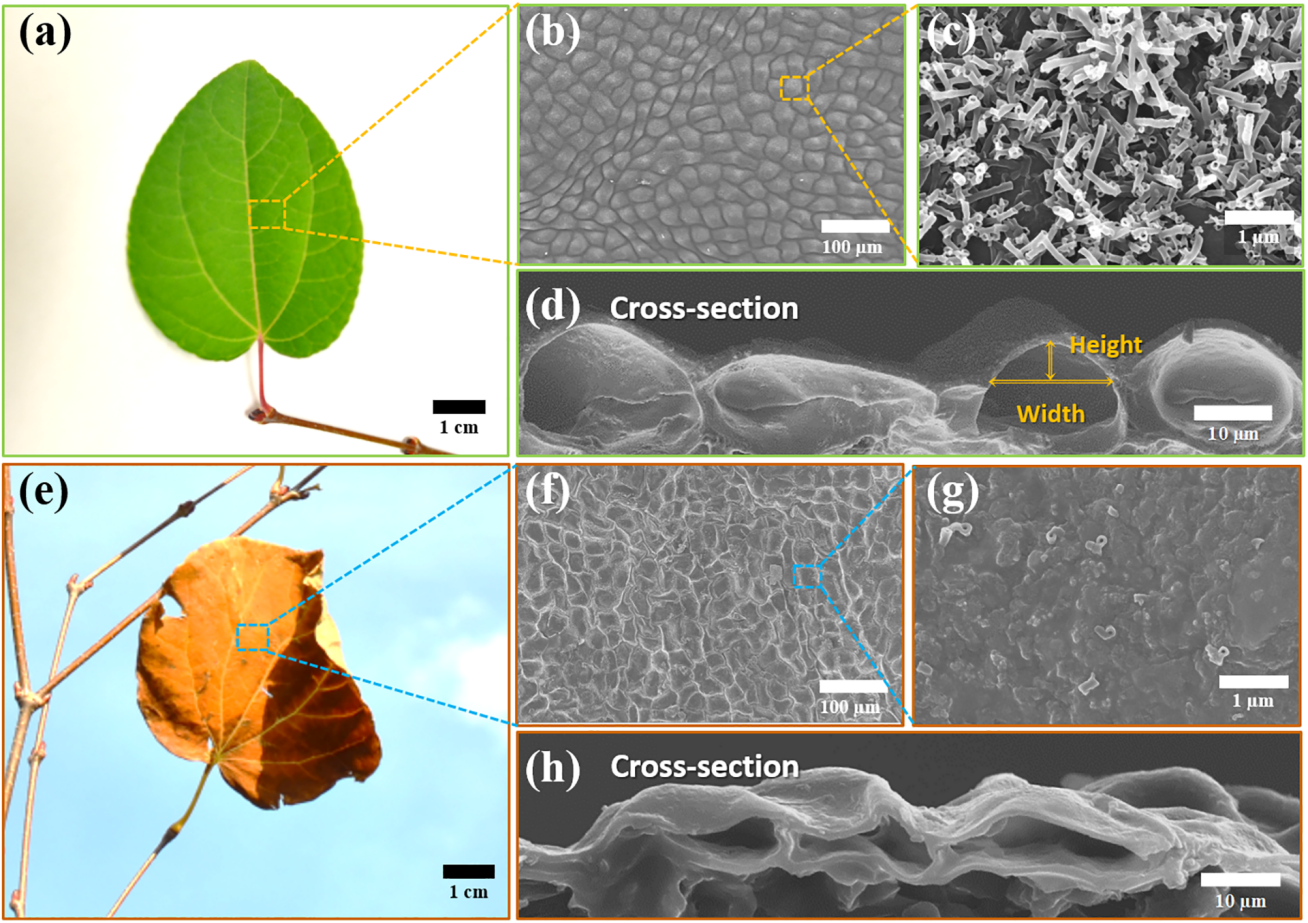
Optical and scanning electron microscope images of a green leaf (a-d) and a brown leaf (e-h). The green leaf (summer) is covered with oblate spheroidal epidermal cells and homogeneous epicuticular waxes. However, on the brown leaf (fall), the epidermal cells are shrunk and the epicuticular waxes are eroded. Magnifications of SEM images: (b, f) = 500x, (c, g) = 50,000x, (d, h) = 2,000x.

Scanning electron microscopy (SEM) images in Fig 2 show the hierarchical, two-tier surface structure of green leaves during the summer (a-d) and brown leaves during the fall (e-h). On the green leaves during summer, microscale papillose epidermal cells form oblate spheroidal bumps as shown in Fig 2(b) and 2(d). The height and width of the epidermal cells in the summer is 8 ± 5 and 25 ± 6 *μ*m, respectively. At the nanoscale, a dense layer of epicuticular wax tubules homogeneously covers the summer leaves as in Fig 2 (c). The epicuticular wax has an elongated tube shape with a diameter of about 100 nm and a length of 1 *μ*m.

In the dry fall, when the water content decreases inside the epidermal cells, the turgor pressure will be reduced [6]. Hence, the microscale epidermal cells with soft walls presumably deflate as the internal turgor pressure decreases. Accordingly, we observed shrinkage of the epidermal cells on a leaf as shown in Fig 2(f) and 2(h). The cell height and width decreased by 47% (4 ± 3*μ*m) and 24% (19 ± 4*μ*m,), respectively. The coverage of epicuticular waxes is significantly reduced especially on top of epidermal bumps in the fall. The SEM image (Fig 2(g)) of a fall leaf shows that the epicuticular wax tubules are eroded and the surface is flattened.

### Wetting state on a leaf

A water droplet on a rough surface can be described by either a Wenzel or Cassie-Baxter state [21, 22]. If a droplet wets the surface (i.e. when no air is trapped between the droplet and the surface), the droplet is in the Wenzel state with an apparent contact angle θ* described as cosθ* = *r* cos*θ* [21]. Here, *r* is the roughness and *θ* is the contact angle for the flat surface of the same material. The roughness is calculated as the ratio of the actual surface area to the apparent (projected) surface area. In the Cassie-Baxter state, a droplet does not completely wet the rough surface (i.e. air is trapped between the droplet and the surface) with an apparent contact angle θ* expressed [22] as cosθ* = *ϕ*_*s*_(cos*θ* + 1) − 1, where *ϕ*_*s*_ is the areal fraction of the substrate that is in contact with the liquid (solid/liquid interface).

Due to geometric complexity in a leaf surface, it is difficult to predict which wetting state occurs over the epidermal bumps. To experimentally characterize the microscopic wetting state between a water interface and a leaf surface, a water droplet was frozen while sitting on the leaf (see S1 Fig). Then, the detached frozen droplet was visualized using environmental scanning electron microscopy (ESEM). We found that the bottom interface of the frozen droplet follows the contours of the microscale epidermal cells (the first-tier rough surface), which indicates the Wenzel state at the microscale. At the nanoscale, the second-tier of surface roughness is assumed to be in the Cassie-Baxter state because water cannot penetrate between the elongated epicuticular wax nanocrystals according to the pressure balance described in “Materials and Methods” section. Therefore, we can assume that droplets on the Katsura leaf exhibit a partial Wenzel state [23, 24]: the Wenzel state for the first-tier roughness at the microscale and the Cassie-Baxter state for the second-tier roughness at the nanoscale.

### Combined Wenzel and Cassie-Baxter contact-angle model

**Fig 3:**
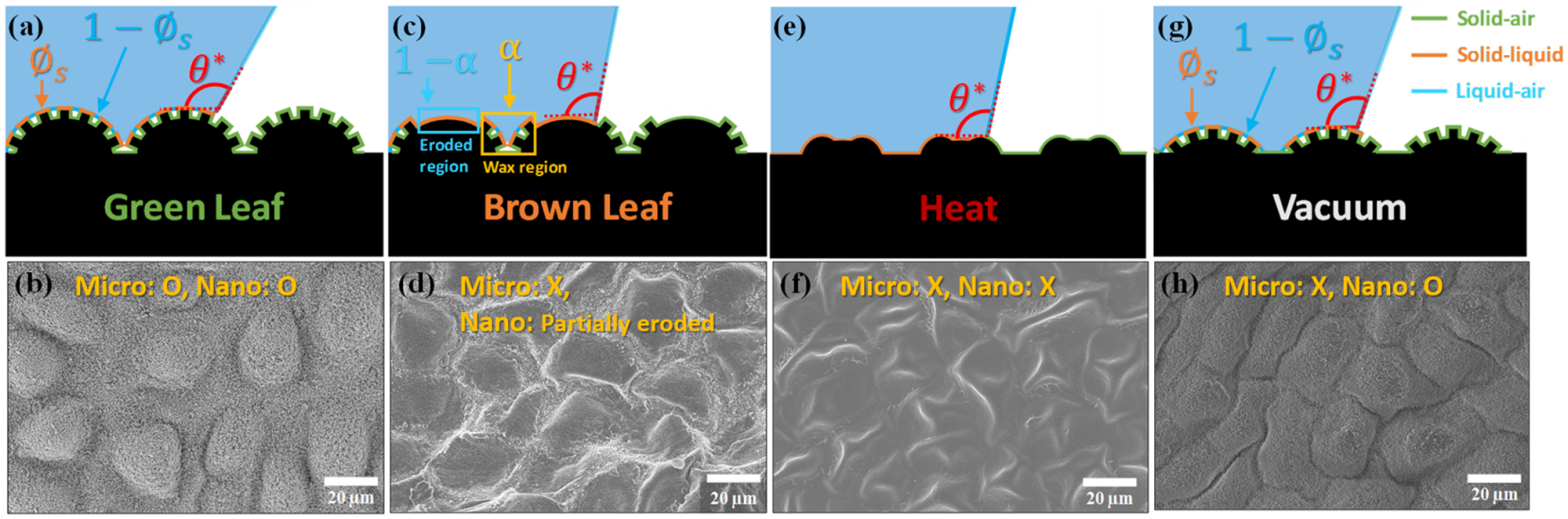
(a,c,e,g) Schematics illustrating wetting states for various leaf conditions; (a) green, (c) brown, (e) heat-treated, and (g) vacuum-treated leaves. Here, θ* is the apparent contact angle, *ϕ*_*s*_ is the areal fraction of solid/liquid interface, and *α* is the intact wax areal fraction. The scanning electron microscope images (b,d,f,h 2,000x) provide examples of the four leaf conditions; (b) green, (d) brown, (f) heat-treated, and (h) vacuum-treated leaves.

To account for two-tier surface roughness, we propose a combined Wenzel and Cassie-Baxter model. Fig 3(a) and 3(c) show schematics for wetting states of green and brown Katsura leaves. Here, the green line represents the solid/air interface, whereas the orange and blue lines represent the solid/liquid and liquid/air interfaces, respectively. By finding the equilibrium state of surface energies (see details in “Supplementary information Eq (1-2)”), the apparent contact angle for green leaves can be expressed as

$$\begin{array}{ccc} & \cos\theta^{*} & =r_{\text{micro}}\;\phi_{\text{nano}}\;\text{cos}\theta -r_{\text{micro}}\left(1-\phi_{\text{nano}}\right), \end{array}$$ (2)

where *r*_micro_ is the roughness of epidermal cells at the microscale, and *ϕ*_nano_ is the liquid/solid interfacial areal fraction on top of wax tubules at the nano scale. The first term on the right hand side is from the solid/liquid areal fraction (*r*_micro_ *ϕ*_nano_), and the second term depends on the liquid/air areal fraction (*r*_micro_(1 − *ϕ*_nano_)). This equation is only valid for green leaves which are uniformly covered by wax tubules.

Brown leaves have partially eroded wax regions, creating an inhomogeneous surface roughness (see S2 Fig). The eroded wax regions presumably do not trap air pockets like the Cassie-Baxter state. Therefore, to capture the effect of the inhomogeneous roughness, we define the areal fraction of intact to eroded wax regions as *α* as shown in S2 Fig. Then, we can modify Eq (2) using the intact area fraction *α* as:

$$\begin{array}{c} \cos\theta^{*}=\alpha \;r_{\text{micro}}\left(\phi_{\text{nano}}\left(\text{cos}\;\theta +1\right)-1\right)+\left(1-\alpha \right)r_{\text{micro}}\;\text{cos}\theta . \end{array}$$ (3)

This generalized equation can describe both green and brown leaves by tuning *α*, e.g. green leaves with *α* = 1 and brown leaves with 0 < *α* < 1.

We experimentally measured all the parameters in (3): *r*_micro_, *ϕ*_nano_, *α*, and *θ*. First, side cross-sectional images are used to determine the surface roughness (*r*_micro_ = 1.16 ± 0.08 for the green leaves (summer); *r*_micro_ = 1.07 ± 0.02 for the brown leaves (fall) as in S3 Fig). Second, the liquid/solid interfacial area fraction of waxes has been measured by field-emission scanning electron microscopy (FE-SEM) (*ϕ*_nano_ = 0.25 ± 0.05 as in S4 Fig). Third, the ratio of the intact to eroded wax areas are quantified from the top-viewed SEM images (*α* = 1 for the green leaves; *α* = 0.46 ± 0.14 for the brown leaves as in S2 Fig). Finally, we measured the contact angle for a flat wax surface, *θ*, to be 101 ± 4° (*θ*_Adv_/*θ*_Rec_ = 117°/86°) by depositing chemically extracted wax on a flat glass substrate (see S5 Fig).

Theoretical values of the apparent contact angle θ* are calculated using the combined Wenzel and Cassie-Baxter model ((3)) and are shown in Table 1. The apparent contact angles of green and brown leaves are estimated to be 158° and 121° respectively. These predicted contact angles are similar to our experimentally measured averaged contact angles of 147 ± 4° ($\theta_{\text{Adv}}^{*}/\theta_{\text{Rec}}^{*}=157{}^{\circ}/137{}^{\circ}$) in the summer, and 123 ± 8 ($\theta_{\text{Adv}}^{*}/\theta_{\text{Rec}}^{*}=151{}^{\circ}/96{}^{\circ}$) in the fall.

**Table 1:** Theoretical and experimental contact angles, roughness, and the areal fraction of intact wax regions in different leaves.

Samples	Theoretical contact angle	Experimental contact angle	Roughness (*r*_micro_)	Intact areal fraction (*α*)
1. Green leaves (Apr.—Jul.)	158°	147 ± 4°	1.16 ± 0.07	1
2. Brown leaves (Nov.—Dec.)	121°	123 ± 8°	1.07 ± 0.02	0.46 ± 0.14
3. Heat treated leaves	103°	106 ± 3°	1.13 ± 0.02	0
4. Vacuum dried leaves	152°	147 ± 5°	1.10 ± 0.03	1

### Heat and vacuum treatments on a leaf

In order to study the effect of micro- and nano-structures on leaf wettability, we treated Katsura leaves in two different conditions: high temperature and vacuum (low pressure). We found that each treatment modifies morphological configurations differently on the micro- and nanoscale features as shown in Fig 3 and S6 Fig. For example, the high-temperature treatment at 65° for 4 hours shrinks and crumples the epidermal cells, and removes the epicuticular nano-wax tubules as shown in Fig 3(f) and S6(a) Fig. Therefore, the intact areal fraction becomes zero, but the roughness stays in a similar range of value by crumpled epidermal cells. When applying the low-pressure treatment, the intact areal fraction remains the same but the epidermal cells are shrunken (see Fig 3(h) and S6(b) Fig).

**Fig 4:**
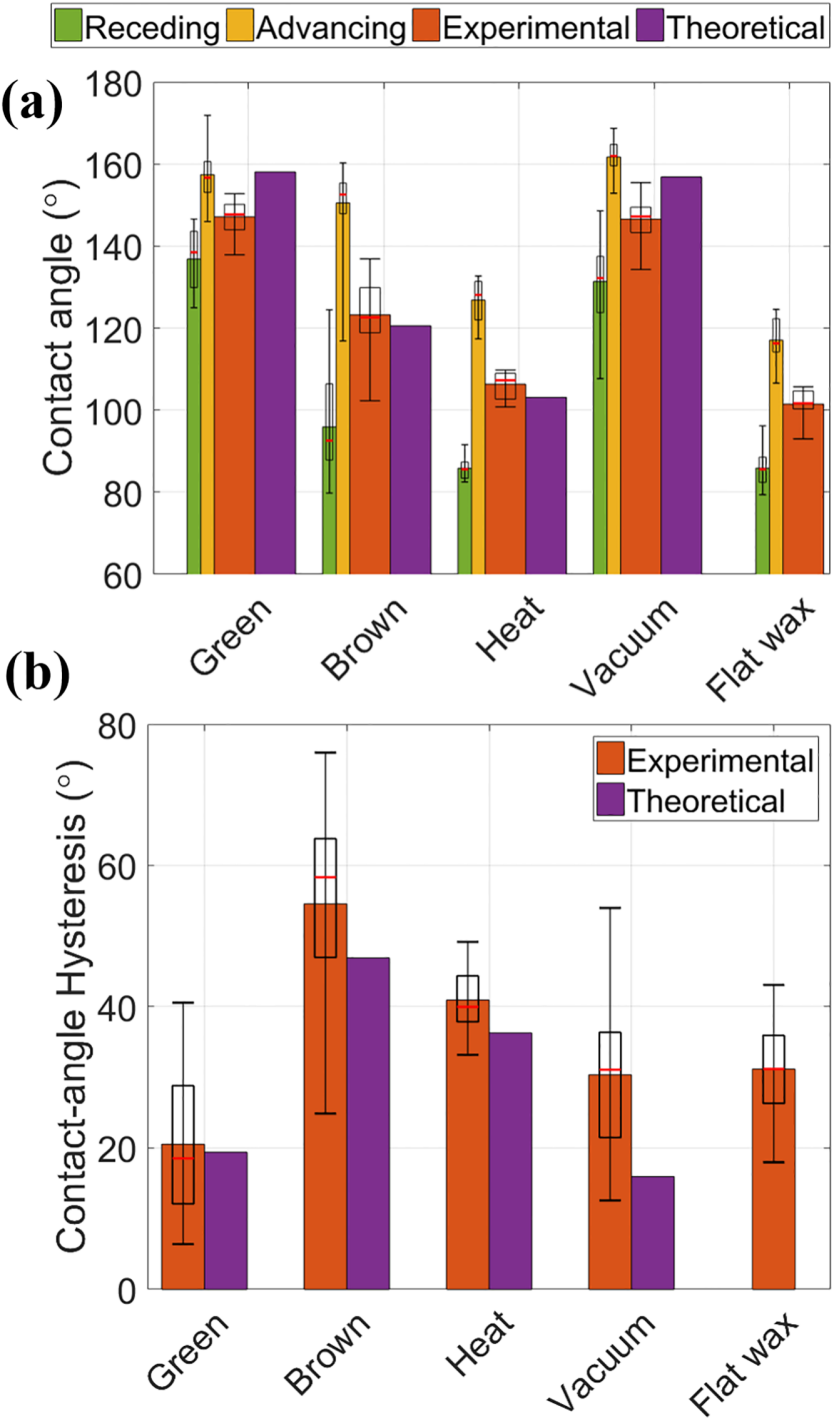
(a) Contact angles of different leaves; green, brown, heat-treated, and vacuum-treated leaves. Receding (green) and advancing (yellow) contact angles are measured on a tilting method. Here, the boxes represent the interquartile range (IQR) between first and third quartiles and the red line inside represents the median. The whiskers denote the lowest and highest values within 1.5 x IQR from the first and third quartiles, respectively. The averaged contact angle (red) is an average of the receding and advancing contact angles. Theoretical apparent contact angles (purple) are calculated from Eq (3). (b) Contact-angle hysteresis of different leaves. The contact angle hysteresis is the difference between the advancing and receding contact angles, which presents the stickiness of water droplets on a leaf. Theoretically Eq (4) predicted contact-angle hysteresis are shown as purple bars.

We compared the computed and experimentally measured contact angles for each type of leaf sample as shown in Fig 4(a) using the combined Wenzel and Cassie-Baxter model. The heat-treated leaves (at 65°C for 4h) had an experimentally averaged contact angle of 106 ± 3° ($\theta_{\text{Adv}}^{*}/\theta_{\text{Rec}}^{*}=127{}^{\circ}/86{}^{\circ}$) and the predicted contact angle of 103°. Here, the temperature and duration of the heat treatment are chosen to ensure that the leaf reaches a steady-state contact angle (see S7 Fig). The vacuum treatment to dehydrate the leaves does not melt the epicuticular waxes (*α* = 1) but does shrink the epidermal cells as shown in Fig 3(h) and S6(b) Fig. For the vacuum treatment, the averaged contact angle was 147 ± 5° ($\theta_{\text{Adv}}^{*}/\theta_{\text{Rec}}^{*}=162{}^{\circ}/131{}^{\circ}$) and the theoretical apparent contact angle was 152°. The experimental and theoretical contact angles for these two types of leaves agree relatively well. Furthermore, we tested leaves without nano-structures (nano-wax tubules), but preserved micro-structures (epidermal cells), and measured an averaged contact angle of 94 ± 4° (112° /77°).

### Contact-angle hysteresis

Contact-angle hysteresis is another important measure, which determines how a water droplet sticks on a surface. We observed that brown leaves have a high contact-angle hysteresis of about 55° while green leaves have only about 20°. As a result, the critical angle for a 10 *μ*L droplet to roll off a leaf changes from 12 ± 6° for green leaves to 70 ± 20° for brown leaves. The contact-angle hysteresis on a leaf is affected by not only the contact angle and its hysteresis on a flat wax-coated surface, but also the geometric properties of the original surface (intact areal fraction (*α*) and roughness (*r*_micro_)) as proposed in [25]. To evaluate the contact-angle hysteresis, we take a derivative of (3) with respect to a small angle difference, i.e. the contact-angle hysteresis on a flat wax surface (Δ*θ*_flat_ ≡ *θ*_Adv_−*θ*_Rec_). Then, the contact-angle hysteresis on a leaf ($\Delta \theta_{\text{leaf}}^{*}\equiv \theta_{\text{Adv}}^{*}-\theta_{\text{Rec}}^{*}$)) can be expressed as

$$\begin{array}{c} \Delta \theta_{\text{leaf}}^{*}\;\sin\theta_{\text{leaf}}^{*}=r_{\text{micro}}\left(\alpha \left(\phi_{\text{nano}}-1\right)+1\right)\;\Delta \theta_{\text{flat}}\;\text{sin}\;\theta_{\text{flat}}\;. \end{array}$$ (4)

Calculated contact-angle hystereses for the green, brown, heat-treated, and vacuum-treated leaves are in good agreement with experimental contact-angle hystereses as shown in Fig 4(b).

Using such a contact-angle change, we can estimate how much water volume leaves can hold. For brown leaves with a higher contact-angle hysteresis, larger dew droplets (or raindrops) can remain pinned to the surface compared to green leaves. This can be quantified by considering a force balance between contact angle hysteresis and gravity [26]:

$$\begin{array}{c} \pi a\gamma_{\text{LA}}\left(\cos\theta_{\text{Rec}}^{*}-\cos\theta_{\text{Adv}}^{*}\right)\approx V_{c}\rho_{\text{water}}g\sin\beta , \end{array}$$ (5)

where *a* is the radius of the droplet’s contact area with the surface, *γ*_LA_ is the surface tension, *V*_*c*_ is the critical volume at which the droplet will slide off the leaf, *ρ*_water_ is the density of water, *g* is gravity, and *β* is the leaf’s tilt angle. Plugging in the geometric relations $a=R\;\text{sin}\;\bar{\theta^{*}}$ (*R*; the radius of the droplet) and $V_{c}=\frac{\pi }{3}R^{3}{\left(1-\text{cos}\;\bar{\theta^{*}}\right)}^{2}\left(2+\text{cos}\;\bar{\theta^{*}}\right)$ and solving for *V*_*c*_, we obtain the expression for the maximum allowable droplet volume on a leaf:

$$\begin{array}{cc} V_{c} & ={\left(\frac{\pi \gamma_{\text{LA}}\text{sin}\;\bar{\theta^{*}}\left(\text{cos}\;\theta_{\text{Rec}}^{*}-\text{cos}\;\theta_{\text{Adv}}^{*}\right)}{\rho_{\text{water}}g\;\text{sin}\;\beta }\right)}^{3/2}\times \\ & {\left(\frac{3}{\pi {\left(1-\text{cos}\;\bar{\theta^{*}}\right)}^{2}\left(2+\text{cos}\;\bar{\theta^{*}}\right)}\right)}^{1/2}. \end{array}$$ (6)

Using (6), we can find the dimensionless ratio of *V*_*c*,brown_/*V*_*c*,green_ by using the experimental contact angle measurements (cf. Fig 1 caption). This calculation reveals that brown leaves can hold droplets that are approximately 17 times larger than droplets on the green leaves. Assuming that the same number of droplets stick on a given leaf (i.e. neglecting variations in weather), the total mass of water on brown leaves can be up to 17 times larger than with green leaves, such that they might experience a higher torque at the base. Hypothetically, such a higher torque helps facilitate detachment and leaf-fall.

## Discussion and conclusions

In this study, we found that Katsura (*Cercidiphyllum japonicum*) leaves change from superhydrophobic in the summer ($\theta_{\text{Adv}}^{*}/\theta_{\text{Rec}}^{*}=157{}^{\circ}/137{}^{\circ}$) to hydrophobic in the fall ($\theta_{\text{Adv}}^{*}/\theta_{\text{Rec}}^{*}=151{}^{\circ}/96{}^{\circ}$). Moreover, the contact-angle hysteresis significantly increases from 20° to 55°. Such wettability changes are induced by alterations in a leaf’s two-tier structure; the microscale epidermal cells shrink and the coverage of nanoscale epicuticular waxes decreases due to the erosion in the fall. This epicuticular wax helps leaves maintain high photosynthesis efficiency through the self-cleaning effect as well as high optical transparency [27–29]. Simultaneously, a number of industrial applications seek similar properties and leaf wax can potentially be used as a sustainable coating material.

To evaluate this idea, a mono-crystalline silicon solar cell (GP80X80-10A100, sunnytech) was coated with waxes extracted from Katsura leaves (see S8(a) and S8(b) Fig). We observed that there is no significant difference in the maximum power output; 0.56 ± 0.02 W for a uncoated solar panel and 0.55 ± 0.02 W for a wax-coated solar cell. In addition, artificial dirt (glass spheres; GL0191B5/90-106, MO-SCI corporation) is easily removed via the self-cleaning effect on a wax-coated solar cell as shown in S8(c) Fig. Despite drawbacks such as the erosion of the wax layer from the surface, Such a sustainable coating material extracted from leaves might find a range of applications including photosynthesis devices as well as various hydrophobic surface treatments.

In this study, leaf samples were assumed to be air-pollutant free due to the high-air quality of Virginia Tech’s campus where they were collected. However, the contact angle and its hysteresis might be affected by the level of contamination [30–32], water temperature [20], tree species [33], and the position in the crown. In future, the effects of air pollution or position in the crown might be interesting questions.

## Supporting information

S1 Fig Schematics of the procedure to experimentally visualize the liquid/solid interface on epidermal cells using environmental scanning electron microscopy (ESEM). (a) A water droplet is placed on the Katsura leaf. (b) We then freeze the water droplet by rapidly decreasing the temperature using liquid nitrogen. There is 9% volume expansion when the phase of water is changed from liquid to ice. However, the volume expansion is a minor effect because the water has been frozen from the bottom. (c) Due to the chamber pressure, the ice slowly detaches from the leaf surface by sublimating of the ice. (d) An ESEM image of detached ice shows the 25 ± 8 *μ*m width and 8 ± 3 *μ*m height of the ice surface, which are close to the leaf’s epidermal cell width (25 ± 6 *μ*m) and height (8 ± 5 *μ*m).
(PDF)

S2 Fig The areal fraction of intact wax regions is measured by using the contrast difference. We ignored intact wax regions of less than 5 *μ*m^2^ and eroded wax regions of less than 22.5 *μ*m^2^ when measuring the *α* value. (a) The schematic of a brown leaf depicts how the wax-coated and eroded regions are located from the side view. (b) A top-viewed SEM image of a brown leaf clearly shows wax-eroded regions as a dark gray color. (c,d) Tracked boundaries of the eroded regions are shown as a blue line.
(PDF)

S3 Fig The cross-sectional ESEM images of leaves after various treatments:(a) green, (b) brown, (c) heat-treated, (d) vacuum-treated leaves. The surface roughness has been measured from the blue lines extracted from a customized Matlab code.
(PDF)

S4 Fig The areal fraction of liquid/solid interface is evaluated from ESEM images by tracking liquid/solid interface. To determine *ϕ*_nano_, the image histogram was used to choose a reliable and systematic parameter in the grayscale. Typically, two peaks were observed in the histogram as shown in the inset of S4 Fig (a). First, the region brighter than the upper peak is presumably where a liquid droplet meets with solid waxes whereas the region darker than the lower peak is presumably where the bottom leaf surface is. The region between the two peaks is wax that was not in contact with the water droplet. Hence, *ϕ*_nano_ was calculated based on an approximated solid/liquid interface, which is brighter than the upper peak in the grayscale histogram. We assumed the water only touches the top of the nano-wax tububles. The blue line represents the tracked liquid/solid interface of the nano-wax tubules. (a) The green leaf has enough wax to maintain air pockets (Cassie-Baxter state). (b) In the wax-eroded regions of the brown leaf, the wax tubules are not able to sustain the air pocket since most of wax tubules have disappeared (Wenzel state).
(PDF)

S5 Fig (a) 10 ml chloroform was dripped onto a green Katsura leaf which was tilted at about 30 degrees from the horizontal axis. Then, the collected wax-dissolved chloroform solution was coated on a target substrate and gently dried with nitrogen gas. (b) An SEM image of the flat wax-coated substrate. The inset shows an optical image of the wax-coated silicon wafer. (c) A contact angle measurement shows that mean, advancing and receding contact angles are 101 ± 4°, 117 ± 5° and, 86 ± 5°, respectively.
(PDF)

S6 Fig Top and cross-sectional SEM images of leaves with various treatments: (a) A heat-treated Katsura leaf shows shrunken epidermal cells and melted epicuticular waxes. (b) A vacuum dried procedure preserves epiculticular waxes, but makes the cells shrink. (c) Chloroform treatment removed only the epicuticular wax. The epidermal cells were intact from the chloroform treatment.
(PDF)

S7 Fig Contact angle vs. heating-time plots for several oven temperatures. As the epicuticular wax tubules melt away due to the heat, the contact angle decreases. (a) The epicuticular wax tubules did not significantly melt even after 20 hours at 55°C. (b) At 65°C, the wax starts to disappear after 1 hour. (c) Most of the wax tubules melted away after 1 hour at 75°C.
(PDF)

S8 Fig To measure the efficiency of a solar cell, we placed a halogen lamp (200W, 120V, LOWEL PRO) at 70 *cm* from the solar cell. (a) An uncoated solar cell (GP80 × 80-10A 100, sunnytech) has a hydrophilic surface (63.66 ± 2.44°) and performs *V*_*oc*_ = 5.73 ± 0.14 V, *I*_*sc*_ = 98.44 ± 1.88 mA, *P*_max_ = 0.56 ± 0.02 W, where *I*_sc_ is the short-circuit current, *V*_oc_ is the open-circuit voltage, and *P*_max_ is the maximum power. (b) A Katsura wax-coated solar cell has become a hydrophobic surface with the contact angle (97.33 ± 3.11°). It performs *V*_*oc*_ = 5.61 ± 0.12 V, *I*_sc_ = 98.17 ± 2.86 mA, *P*_max_ = 0.55 ± 0.02 W output. (c) Demonstration of self-cleaning effect as a droplet rolls and clean the surface by collecting dirt (white glass spheres with a diameter of 90-106 *μ*m; GL0191B5/90-106 from MO-SCI corporation).
(PDF)

S1 File Combined Wenzel and Cassie-Baxter model. The expression of the apparent contact angle by finding the equilibrium state of surface energies at the three-phase contact line.
(PDF)
