# Correction: Seasonal changes in morphology govern wettability of Katsura leaves

**DOI:** 10.1371/journal.pone.0207425

**Published:** 2018-11-08

**Authors:** Hosung Kang, Philip M. Graybill, Sara Fleetwood, Jonathan B. Boreyko, Sunghwan Jung

There are errors in the Funding section. The correct funding information is as follows: This work was supported by https://www.nsf.gov/awardsearch/showAward?AWD_ID=1604424. Hosung Kang was supported by the Postdoctoral Research Program of Sungkyunkwan University (2015) and Philip M. Graybill was supported by Virginia Tech's BIOTRANS IGEP. The funders had no role in study design, data collection and analysis, decision to publish, or preparation of the manuscript.
