# Supplementary material for: Seasonal changes in morphology govern wettability of Katsura leaves
Source: PLoS One. 2018 Sep 27;13(9):e0202900. doi: 10.1371/journal.pone.0202900 (PMC6159866; doi:10.1371/journal.pone.0202900)
Supplement: S1 Fig — (a) A water droplet is placed on the Katsura leaf. (b) We then freeze the water droplet by rapidly decreasing the temperature using liquid nitrogen. There is 9% volume expansion when the phase of water is changed from liquid to ice. However, the volume expansion is a minor effect because the water has been frozen from the bottom. (c) Due to the chamber pressure, the ice slowly detaches from the leaf surface by sublimating of the ice. (d) An ESEM image of detached ice shows the 25 ± 8 μm width and 8 ± 3 μm height of the ice surface, which are close to the leaf’s epidermal cell width (25 ± 6 μm) and height (8 ± 5 μm). (PDF) [file pone.0202900.s001.pdf]

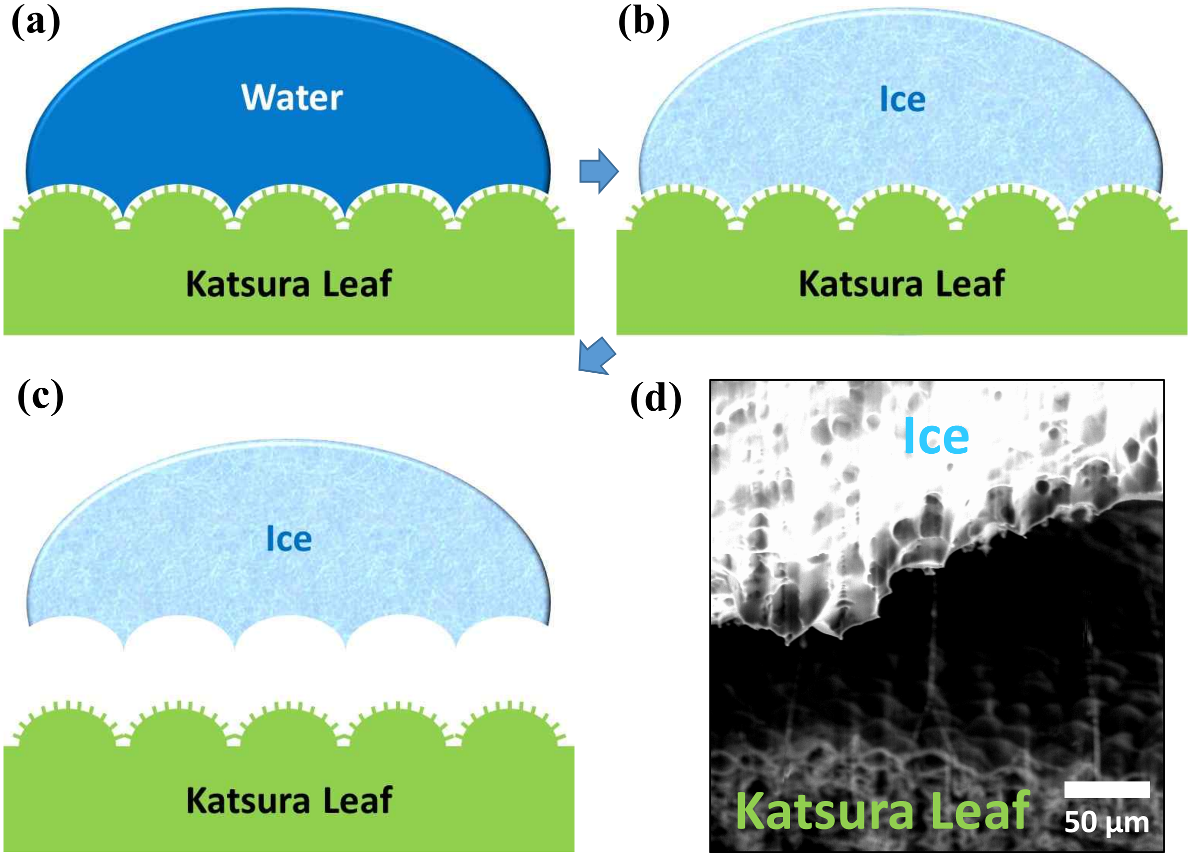

Figure S1: Schematics of the procedure to experimentally visualize the liquid/solid interface on epidermal cells using environmental scanning electron microscopy (ESEM). (a) A water droplet is placed on the Katsura leaf. (b) We then freeze the water droplet by rapidly decreasing the temperature using liquid nitrogen. There is 9 % volume expansion when the phase of water is changed from liquid to ice. However, the volume expansion is a minor effect because the water has been frozen from the bottom. (c) Due to the chamber pressure, the ice slowly detaches from the leaf surface by sublimating of the ice. (d) An ESEM image of detached ice shows the 25.1 ± 7.7 μm width and 7.8 ± 3.1 μm height of the ice surface, which are close to the leaf's epidermal cell width (24.8 ± 6.4 μm) and height (8.1 ± 5.2 μm).
