# Supplementary material for: Seasonal changes in morphology govern wettability of Katsura leaves
Source: PLoS One. 2018 Sep 27;13(9):e0202900. doi: 10.1371/journal.pone.0202900 (PMC6159866; doi:10.1371/journal.pone.0202900)
Supplement: S2 Fig — We ignored intact wax regions of less than 5 μm2 and eroded wax regions of less than 22.5 μm2 when measuring the α value. (a) The schematic of a brown leaf depicts how the wax-coated and eroded regions are located from the side view. (b) A top-viewed SEM image of a brown leaf clearly shows wax-eroded regions as a dark gray color. (c,d) Tracked boundaries of the eroded regions are shown as a blue line. (PDF) [file pone.0202900.s002.pdf]

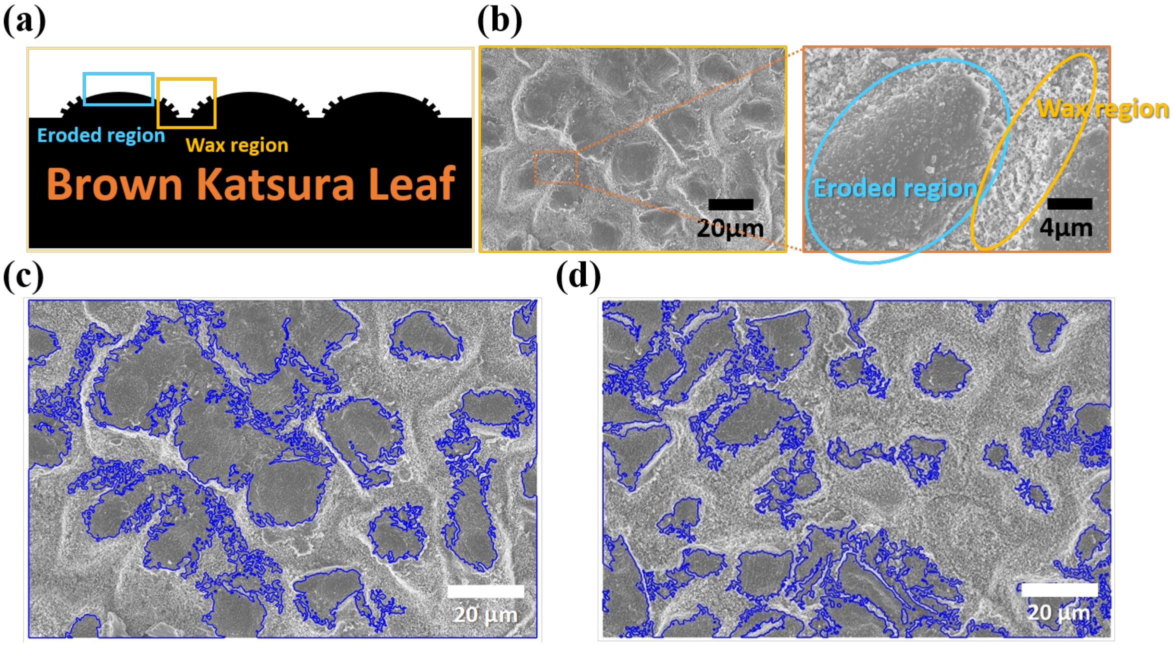

Figure S2: The areal fraction of intact wax regions is measured by using the contrast difference. We ignored intact wax regions of less than 5 μm^2 and eroded wax regions of less than 22.5 μm^2 when measuring the α value. (a) The schematic of a brown leaf depicts how the wax-coated and eroded regions are located from the side view. (b) A top-viewed SEM image of a brown leaf clearly shows wax-eroded regions as a dark gray color. (c,d) Tracked boundaries of the eroded regions are shown as a blue line.
