# Supplementary material for: Seasonal changes in morphology govern wettability of Katsura leaves
Source: PLoS One. 2018 Sep 27;13(9):e0202900. doi: 10.1371/journal.pone.0202900 (PMC6159866; doi:10.1371/journal.pone.0202900)
Supplement: S3 Fig — The surface roughness has been measured from the blue lines extracted from a customized Matlab code. (PDF) [file pone.0202900.s003.pdf]

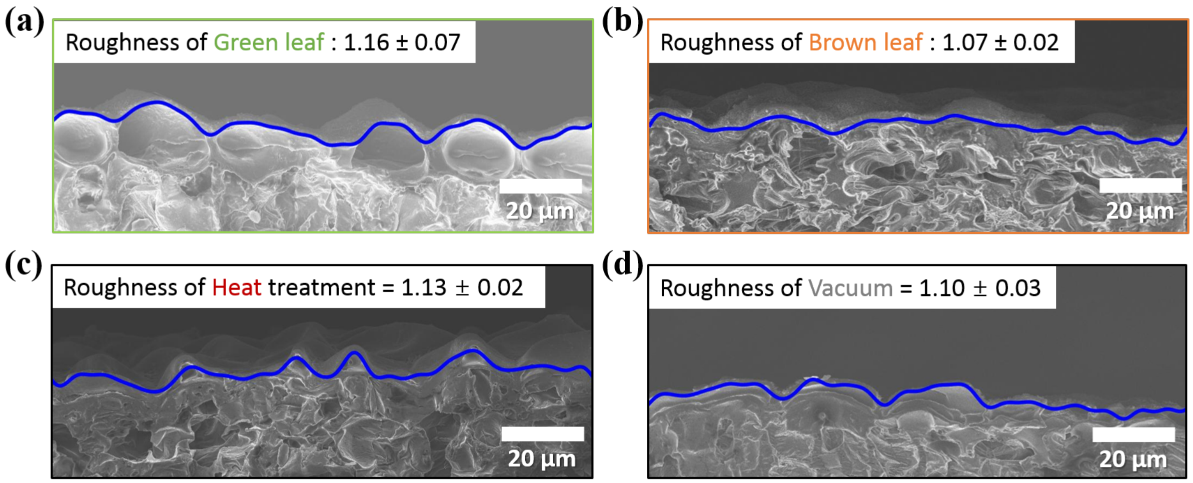

Figure S3: The cross-sectional ESEM images of leaves after various treatments:(a) green, (b) brown, (c) heat-treated, (d) vacuum-treated leaves. The surface roughness has been measured from the blue lines extracted from a customized Matlab code.
