# Supplementary material for: Seasonal changes in morphology govern wettability of Katsura leaves
Source: PLoS One. 2018 Sep 27;13(9):e0202900. doi: 10.1371/journal.pone.0202900 (PMC6159866; doi:10.1371/journal.pone.0202900)
Supplement: S4 Fig — To determine ϕnano, the image histogram was used to choose a reliable and systematic parameter in the grayscale. Typically, two peaks were observed in the histogram as shown in the inset of S4 Fig (a). First, the region brighter than the upper peak is presumably where a liquid droplet meets with solid waxes whereas the region darker than the lower peak is presumably where the bottom leaf surface is. The region between the two peaks is wax that was not in contact with the water droplet. Hence, ϕnano was calculated based on an approximated solid/liquid interface, which is brighter than the upper peak in the grayscale histogram. We assumed the water only touches the top of the nano-wax tububles. The blue line represents the tracked liquid/solid interface of the nano-wax tubules. (a) The green leaf has enough wax to maintain air pockets (Cassie-Baxter state). (b) In the wax-eroded regions of the brown leaf, the wax tubules are not able to sustain the air pocket since most of wax tubules have disappeared (Wenzel state). (PDF) [file pone.0202900.s004.pdf]

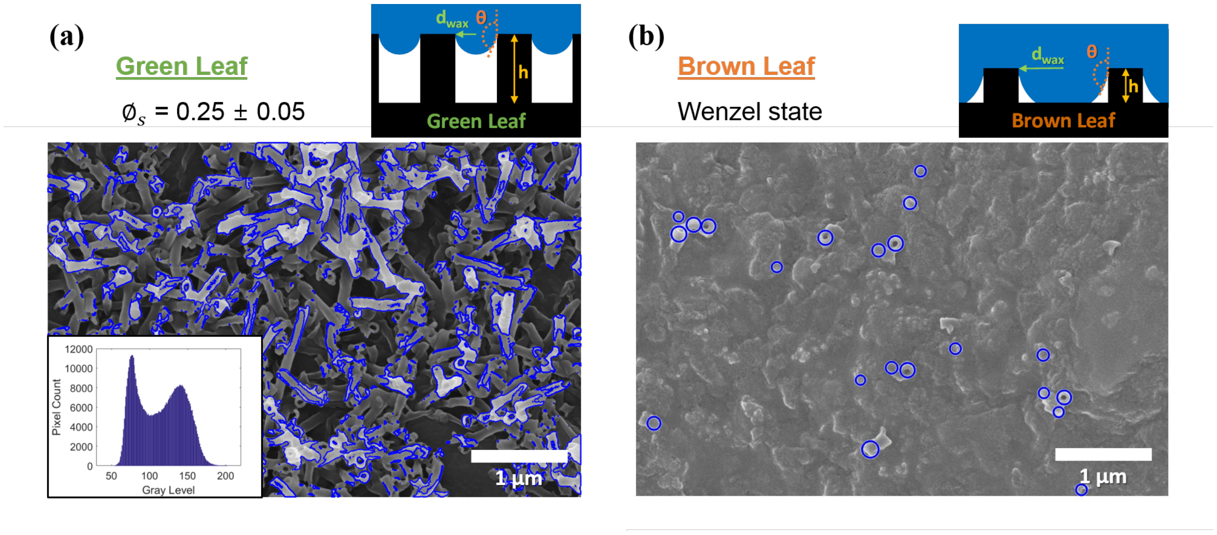

Figure S4: The areal fraction of liquid/solid interface is evaluated from ESEM images by tracking liquid/solid interface. To determine ϕ_{nano}, the image histogram was used to choose a reliable and systematic parameter in the grayscale. Typically, two peaks were observed in the histogram as shown in the inset of FIg. S4 (a). First, the region brighter than the upper peak is presumably where a liquid droplet meets with solid waxes whereas the region darker than the lower peak is presumably where the bottom leaf surface is. The region between the two peaks is wax that would not in contact with the water droplet. Hence, ϕ_{nano} was calculated based on an approximated solid/liquid interface, which is brighter than the upper peak in the grayscale histogram. We assumed the water only touches the top of the nano-wax tububles. The blue line represents the tracked liquid/solid interface of the nano-wax tubules. (a) The green leaf has enough wax to maintain air pockets (Cassie-Baxter state). (b) In the wax-eroded regions of the brown leaf, the wax tubules are not able to sustain the air pocket since most of wax tubules has disappeared (Wenzel state).
