# Supplementary material for: Seasonal changes in morphology govern wettability of Katsura leaves
Source: PLoS One. 2018 Sep 27;13(9):e0202900. doi: 10.1371/journal.pone.0202900 (PMC6159866; doi:10.1371/journal.pone.0202900)
Supplement: S5 Fig — (a) 10 ml chloroform was dripped onto a green Katsura leaf which was tilted at about 30 degrees from the horizontal axis. Then, the collected wax-dissolved chloroform solution was coated on a target substrate and gently dried with nitrogen gas. (b) An SEM image of the flat wax-coated substrate. The inset shows an optical image of the wax-coated silicon wafer. (c) A contact angle measurement shows that mean, advancing and receding contact angles are 101 ± 4°, 117 ± 5° and, 86 ± 5°, respectively. (PDF) [file pone.0202900.s005.pdf]

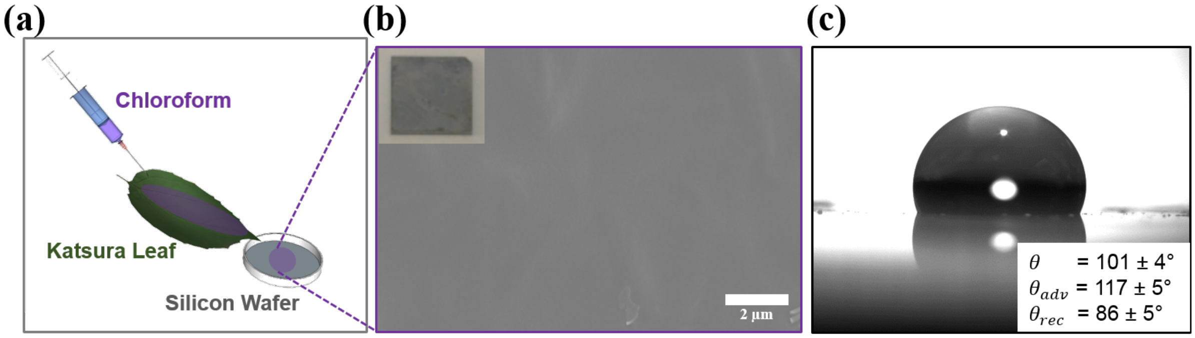


Figure S5: (a) 10 ml chloroform was dripped onto a green Katsura leaf which was tilted at about 30 degrees from the horizontal axis. Then, the collected wax-dissolved chloroform solution was coated on a target substrate and then gently dried with nitrogen gas. (b) An SEM image of the flat wax-coated substrate. The inset shows an optical image of the wax-coated silicon wafer. (c) A contact angle measurement shows that mean, advancing and receding contact angles are 101 $\pm$ 4°, 117 $\pm$ 5°and, 86 $\pm$ 5°, respectively.
