# Supplementary material for: Seasonal changes in morphology govern wettability of Katsura leaves
Source: PLoS One. 2018 Sep 27;13(9):e0202900. doi: 10.1371/journal.pone.0202900 (PMC6159866; doi:10.1371/journal.pone.0202900)
Supplement: S6 Fig — Top and cross-sectional SEM images of leaves with various treatments: (a) A heat-treated Katsura leaf shows shrunken epidermal cells and melted epicuticular waxes. (b) A vacuum dried procedure preserves epiculticular waxes, but makes the cells shrink. (c) Chloroform treatment removed only the epicuticular wax. The epidermal cells were intact from the chloroform treatment. (PDF) [file pone.0202900.s006.pdf]

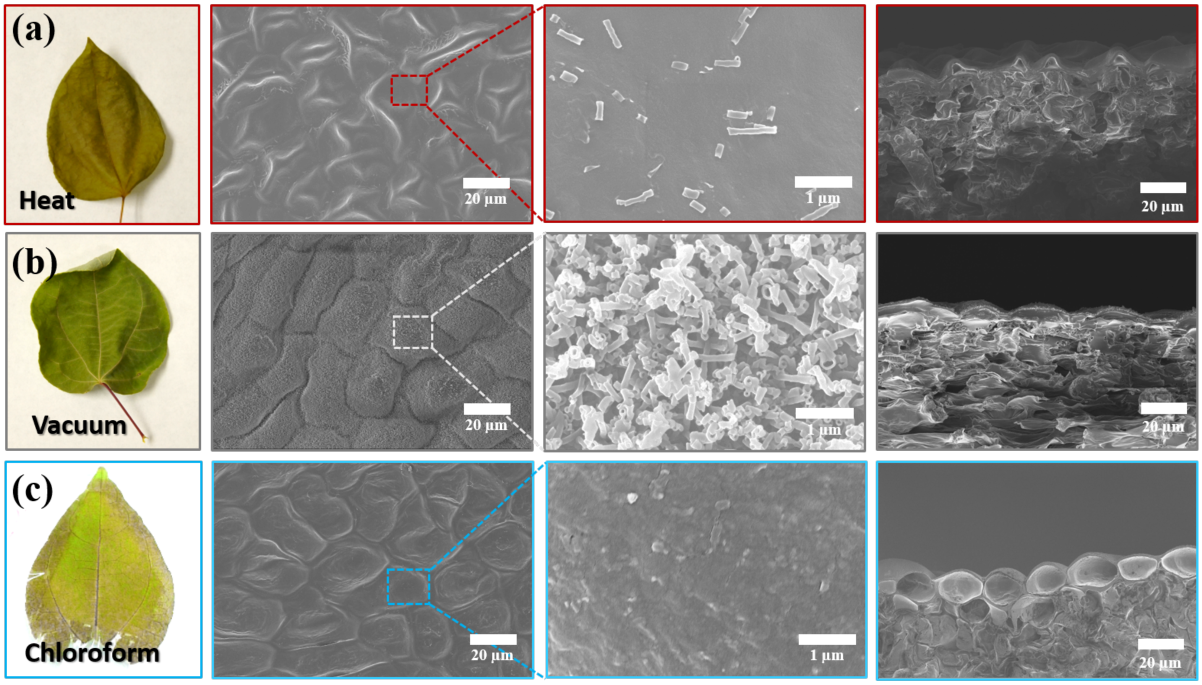

Figure S6: Top and cross-sectional SEM images of leaves with various treatments: (a) A heat-treated Katsura leaf shows shrunken epidermal cells and melted epicuticular waxes. (b) A vacuum dried procedure preserves epiculticular waxes, but makes the cells shrink. (c) Chloroform treatment removed only the epicuticular wax. The epidermal cells were intact from the chloroform treatment.
