# Supplementary material for: Seasonal changes in morphology govern wettability of Katsura leaves
Source: PLoS One. 2018 Sep 27;13(9):e0202900. doi: 10.1371/journal.pone.0202900 (PMC6159866; doi:10.1371/journal.pone.0202900)
Supplement: S7 Fig — As the epicuticular wax tubules melt away due to the heat, the contact angle decreases. (a) The epicuticular wax tubules did not significantly melt even after 20 hours at 55°C. (b) At 65°C, the wax starts to disappear after 1 hour. (c) Most of the wax tubules melted away after 1 hour at 75°C. (PDF) [file pone.0202900.s007.pdf]

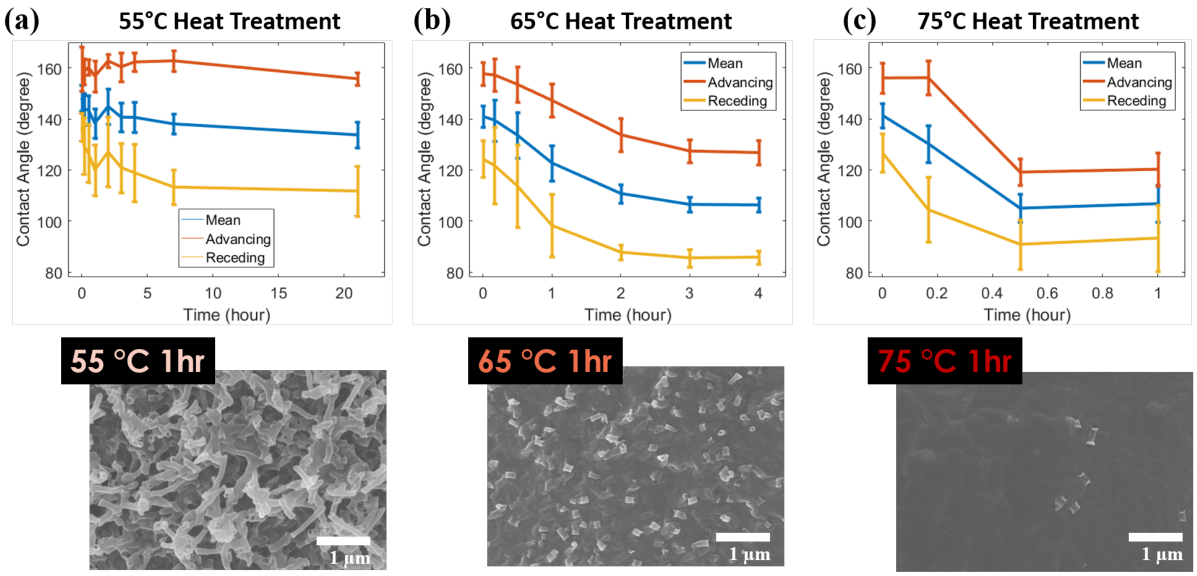

Figure S7: Contact angle vs. heating-time plots for several oven temperatures. As the epicuticular wax tubules melt away due to the heat, the contact angle decreases. (a) The epicuticular wax tubules did not significantly melt even after 20 hours at 55°C. (b) At 65°C, the wax starts to disappear after 1 hour. (c) Most of the wax tubules melted away after 1 hour at 75°C.
