# Supplementary material for: Seasonal changes in morphology govern wettability of Katsura leaves
Source: PLoS One. 2018 Sep 27;13(9):e0202900. doi: 10.1371/journal.pone.0202900 (PMC6159866; doi:10.1371/journal.pone.0202900)
Supplement: S8 Fig — (a) An uncoated solar cell (GP80 × 80-10A 100, sunnytech) has a hydrophilic surface (63.66 ± 2.44°) and performs Voc = 5.73 ± 0.14 V, Isc = 98.44 ± 1.88 mA, Pmax = 0.56 ± 0.02 W, where Isc is the short-circuit current, Voc is the open-circuit voltage, and Pmax is the maximum power. (b) A Katsura wax-coated solar cell has become a hydrophobic surface with the contact angle (97.33 ± 3.11°). It performs Voc = 5.61 ± 0.12 V, Isc = 98.17 ± 2.86 mA, Pmax = 0.55 ± 0.02 W output. (c) Demonstration of self-cleaning effect as a droplet rolls and clean the surface by collecting dirt (white glass spheres with a diameter of 90-106 μm; GL0191B5/90-106 from MO-SCI corporation). (PDF) [file pone.0202900.s008.pdf]

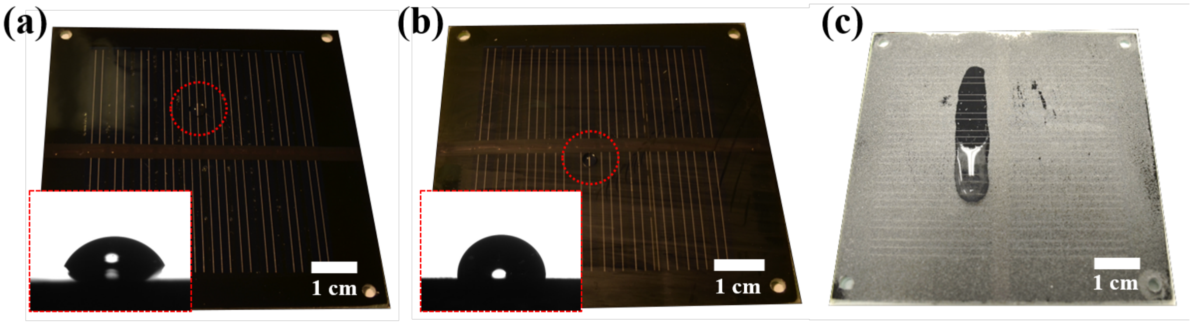

Figure S8: To measure the efficiency of a solar cell, we placed a halogen lamp (200W, 120V, LOWEL PRO) at 70 cm from the solar cell. (a) An uncoated solar cell (GP80$\times$80-10A 100, sunnytech) has a hydrophilic surface (63.66 $\pm$ 2.44°) and performs V_{oc} = 5.73 $\pm$ 0.14 V, I_{sc} = 98.44 $\pm$ 1.88 mA, P_{max} = 0.56 $\pm$ 0.02 W, where I_{sc} is the short-circuit current, V_{oc} is the open-circuit voltage, and P_{max} is the maximum power. (b) A Katsura wax-coated solar cell has become a hydrophobic surface with the contact angle (97.33 $\pm$ 3.11°). It performs V_{oc} = 5.61 $\pm$ 0.12 V, I_{sc} = 98.17 $\pm$ 2.86 mA, P_{max} = 0.55 $\pm$ 0.02 W output. (c) Demonstration of self-cleaning effect as a droplet rolls and clean the surface by collecting dirt (white glass spheres with a diameter of 90-106 μm; GL0191B5/90-106 from MO-SCI corporation).
