# Supplementary material for: Seasonal changes in morphology govern wettability of Katsura leaves
Source: PLoS One. 2018 Sep 27;13(9):e0202900. doi: 10.1371/journal.pone.0202900 (PMC6159866; doi:10.1371/journal.pone.0202900)
Supplement: S1 File — The expression of the apparent contact angle by finding the equilibrium state of surface energies at the three-phase contact line. (PDF) [file pone.0202900.s009.pdf]

Combined Wenzel and Cassie-Baxter model

For the surface with two-tier roughness like green or brown leaves, we express the apparent contact angle using a force balance at the three-phase contact line as

$$\gamma_{\rm LA}\cos\theta^* = r_{\rm micro}\gamma_{\rm SA} - \\ r_{\rm micro}(\phi_{\rm nano}\gamma_{\rm SL} + (1-\phi_{\rm nano})\gamma_{\rm LA} + (1-\phi_{\rm nano})\gamma_{\rm SA})\,, \tag{1}$$

where $\gamma_{\rm LA}$, $\gamma_{\rm SA}$, and $\gamma_{\rm SL}$ are the surface energies on the liquid/air, air/solid, solid/liquid interfaces, respectively. Then, the apparent contact angle becomes

$$\cos\theta^* \quad = r_{\rm micro}\,\phi_{\rm nano}(\cos\theta + 1) - r_{\rm micro}, \tag{2}$$

by replacing $(\gamma_{\rm SA} - \gamma_{\rm SL})/\gamma_{\rm LA}$ with $\cos\theta$.
